# Machine learning-based modeling and analysis of solubility behavior in supercritical CO₂ systems under different operating conditions

**DOI:** 10.3389/fmed.2026.1957061

**Published:** 2026-09-08

**Authors:** Ahmed B. Khoshaim

**Affiliations:** Department of Mechanical Engineering, Faculty of Engineering, King Abdulaziz University, Jeddah, Saudi Arabia

**Keywords:** computations, decision tree regression, ensemble methods, machine learning, tabu search

## Abstract

This study explores the analysis of Nystatin solubility and the density of supercritical carbon dioxide (SC-CO_2_) in supercritical processing. A total of 28 experimental observations were initially collected, which were randomly divided into training (80%) and test (20%) subsets for model development and evaluation, respectively. Output variables include SC-CO_2_ density and solubility of Nystatin, while input parameters include temperature and pressure. Four tree-based machine learning models, namely Random Forest (RF), Extremely Randomized Trees (ET), Gradient Boosting (GB), and XGBoost (XGB) were employed to predict these output variables. For hyper-parameter tuning, the Tabu Search (TS) algorithm was used. For the prediction of Nystatin solubility, the models exhibited commendable performance. Gradient Boosting (GB) outperformed others with an R^2^ value of 0.98142, demonstrating a high level of accuracy in predicting solubility. It also achieved the lowest MAPE and RMSE, indicating superior predictive capabilities. In the case of SC-CO_2_ density prediction, Random Forest (RF) and Extremely Randomized Trees (ET) models demonstrated strong performance with R^2^ of 0.9375 and 0.95155, respectively. Overall, this research provides valuable insights into the estimation of solubility of Nystatin and the density of SC-CO_2_ under varying temperature and pressure conditions. Specifically, machine learning models, specifically GB for solubility and RF and ET for density, has been demonstrated as valuable tools in the prediction of these significant properties.

## 1 Introduction

Processing of solid-dosage drugs with supercritical solvents such as CO_2_ has been on the focus of attention recently owing to the several benefits of this type of process. The process can be regarded as green manufacturing, due to the lack of organic solvents in the process. Moreover, the process can result in preparation of nanosized medicines (oral solid dosage), which have higher solubility compared to the original micron-sized drug particles (1–3). From the physical and thermodynamic point of view, when the particle's size reduces, its free surface energy would enhance and then result in enhanced solubility in aqueous solutions. Considering the fact that the low solubility of drugs in aqueous solutions is a major problem for pharmaceutical sector, this type of process would be of great beneficial for green manufacture of nanosized drug particles in the form of oral solid dosage (4–6).

Different aspects of supercritical processing of drugs can be studied among which the solubility is important which needs to be well studied in order to find its relationship with pressure and temperature. Efficient computation of small-molecular pharmaceutical solubility has become increasingly important in the pharmaceutical industry because solubility is a key determinant of drug absorption, formulation performance, and therapeutic efficacy. Reliable estimation of solubility enables researchers to evaluate the behavior of pharmaceutical compounds under various processing and physiological conditions without relying exclusively on time-consuming and resource-intensive experimental studies. Computational prediction methods facilitate the rapid screening of drug candidates, support the selection of appropriate formulation strategies, and assist in identifying optimal processing conditions during product development (7).

Basically, computational techniques are cost effective for application in determination of drug solubility in supercritical solvents (8–11). For the empirical and semi-empirical models, some experimental data are required to be collected, and the methods can be adopted to estimate the solubility for a wide range of operational parameters (12, 13). Indeed, the models can be used to save time and cost of experiments for drug nanonization in supercritical processing. Some recent works proved that the models based on machine learning are superior for correlation of drug solubility data in supercritical CO_2_, and the relationship between solubility and pressure/temperature can be well understood using these models (4, 14). Different models based on machine learning integrated to optimization algorithms have been developed for correlation of solubility data for small-molecule drugs.

The rapid advancement of machine learning (ML) has significantly expanded the capabilities of data-driven prediction, making it an indispensable methodology for solving complex regression problems across scientific and engineering disciplines (7, 15). Regression-based ML algorithms are designed to establish quantitative relationships between input variables and continuous target responses, providing an effective framework for modeling nonlinear systems that are difficult to describe using conventional analytical approaches (16). Among the available regression techniques, ensemble learning methods have attracted considerable attention because they improve predictive performance by integrating the outputs of multiple base learners rather than relying on a single predictive model. Decision tree-based ensemble algorithms are particularly effective owing to their ability to capture intricate nonlinear relationships, reduce prediction variance, and enhance model generalization through collaborative learning strategies (17, 18).

In the present study, four tree-based ensemble regression algorithms—Gradient Boosting (GB), Extreme Gradient Boosting (XGBoost), Random Forest (RF), and Extremely Randomized Trees (ET)—were developed to model the solubility of nystatin in supercritical carbon dioxide (SC-CO₂). To further improve model performance, the hyperparameter optimization process was carried out using the Tabu Search (TS) algorithm, which systematically explores the search space to identify optimal parameter combinations. To the best of our knowledge, this is the first study to integrate these ensemble learning algorithms with TS optimization for predicting the solubility behavior of Nystatin in SC-CO₂, providing an accurate and efficient computational framework for pharmaceutical solubility modeling.

This research has significantly advanced our understanding of Nystatin solubility variations in supercritical processing of drugs. It has also provided valuable insights into the performance and suitability of four machine learning models (RF, ET, GB, XGB) in predicting density of SC-CO_2_ and solubility of Nystatin drug in the solvent. Additionally, the application of Tabu Search for hyper-parameter optimization has further refined the predictive capabilities of these ensemble models, offering valuable tools for various fields and industries. Accordingly, the novelty of this study lies in developing optimized machine-learning models for accurately predicting Nystatin solubility across different pressure and temperature conditions.

## 2 Dataset

The dataset of drug solubility for this work was sourced from reference (19), consists of solubility data entries for the drug namely Nystatin. A total of 28 experimental observations were collected for model development, which were randomly divided into training (80%) and test (20%) subsets. Each entry represents a distinct set of values, encompassing temperature, pressure, density, and the solubility of Nystatin (11). The dataset employed in this study is identical to that previously used by Meng and Liu (11) and Zhang (16) for machine-learning-based solubility analysis of Nystatin. Accordingly, the principal methodological framework and analytical procedures established in their works were adopted and implemented in the present investigation. The supercritical condition for CO_2_ is at pressure of 7.38 MPa and the temperature of 304.13 K. The collected data are beyond this point to ensure the supercritical state for the solvent. The solubility measurements were conducted over pressure and temperature ranges of 12–30 MPa and 308–338 K, respectively, using supercritical CO₂ solvent.

## 3 Methodology

### 3.1 Tabu search (TS)

Tabu Search is a powerful optimization technique employed to tackle complex combinatorial optimization problems. This method is rooted in the concept of maintaining a dynamic “tabu” list, which tracks recent actions and guides the search process. At its essence, the algorithm functions by maintaining a log of “tabu” actions (7, 20, 21).

**Figure 1:**
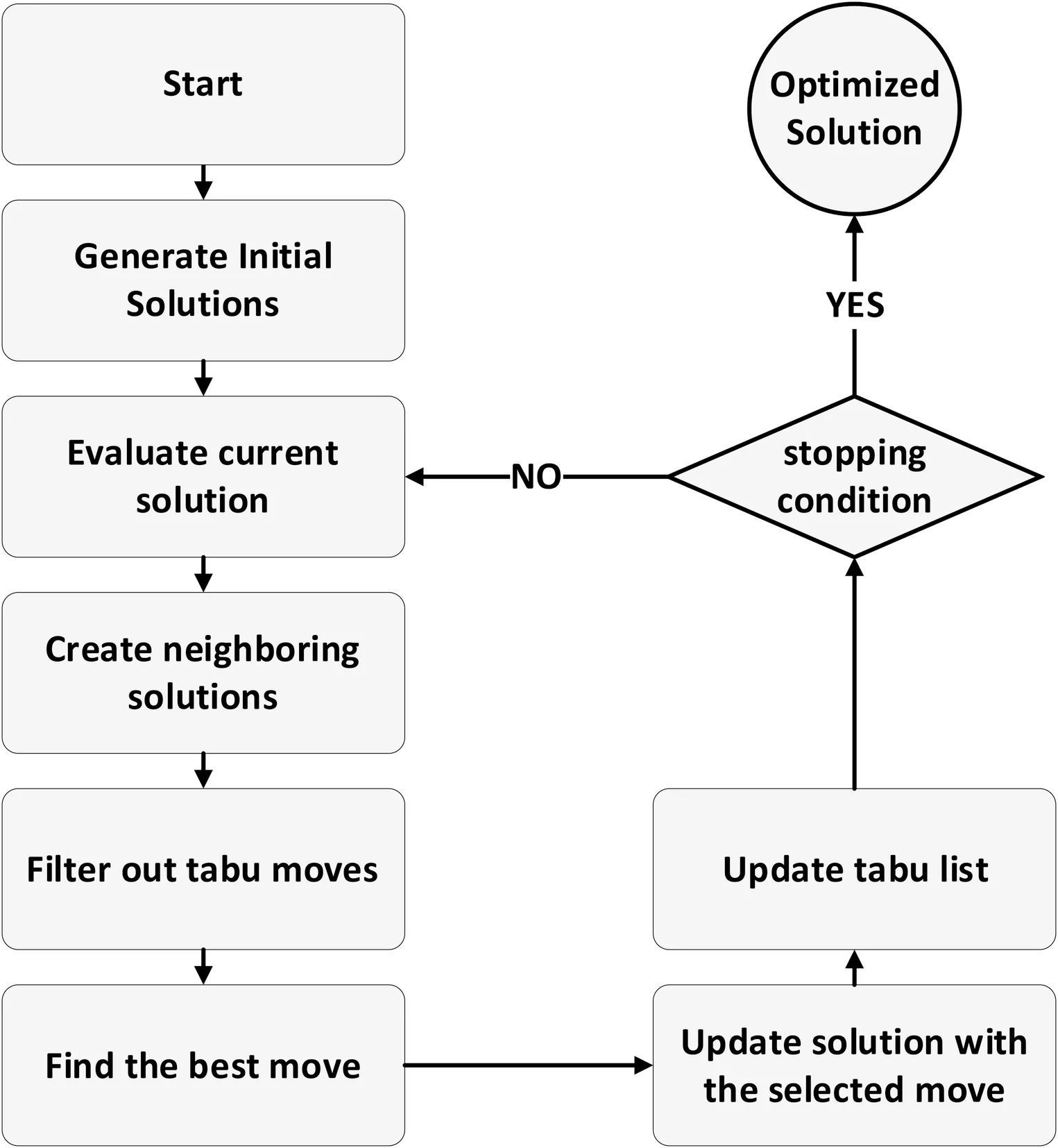
Flowchart of tabu search algorithm.

TS algorithm begins by generating an initial feasible solution, which serves as the starting point for an iterative optimization process. During each iteration, a neighborhood of candidate solutions is explored to identify the most promising alternative according to the optimization objective. The selection of the subsequent solution is determined by both its ability to improve the objective (fitness) function and its adherence to the tabu restrictions, which prevent recently visited solutions or search directions from being repeatedly selected (7). By maintaining this adaptive memory mechanism, the algorithm effectively avoids local optima and encourages a more comprehensive exploration of the search space. The optimization procedure is repeated until a predefined termination criterion is satisfied, such as achieving a target objective value, reaching the maximum allowable number of iterations, or meeting another specified convergence condition (22, 23). The procedural steps undertaken by this algorithm are visually depicted in Figure 1 (24).

### 3.2 Decision tree (DT) regression

The Decision Tree (DT) Regression is a versatile machine learning algorithm employed for tasks encompassing both classification and regression. Unlike traditional linear regression models, DT Regression can capture non-linear associations between input features and the output parameter, making it a valuable tool in data analysis and predictive modeling (25).

The Mean Squared Error (MSE) is a common cost function used in regression analysis, and its mathematical definition is as follows:

$$MSE=\frac{1}{n}\overset{n}{\underset{i=1}{\sum }}(y_{i}-\hat{y_{i}}{)}^{2}$$

where *n* stands for the size of training subset, $y_{i}$ denotes the observed target value for the *i*-th data sample, and $\hat{y_{i}}$ stands for the estimated value for the same sample.

### 3.3 Ensemble regression models based on decision trees

Decision-tree-based ensemble learning methods have gained considerable attention in regression modeling due to their ability to enhance predictive accuracy by aggregating the predictions of multiple individual learners into a unified modeling framework. Rather than relying on a single decision tree, these approaches generate a collection of diverse learners whose combined predictions produce greater accuracy, stability, and generalization capability. Among the most widely used tree-based ensemble algorithms are Random Forest (RF), Extremely Randomized Trees (Extra Trees, ET), Gradient Boosting (GB), and Extreme Gradient Boosting (XGBoost). Although all four methods employ decision trees as their fundamental learning units, they differ in the way individual trees are constructed and aggregated, enabling them to address a broad range of nonlinear regression problems with high predictive efficiency (26).

Random Forest (RF) is a bagging-based ensemble algorithm that enhances prediction reliability by combining the outputs of numerous decision trees. Each tree is independently trained using a bootstrap sample randomly drawn from the original training dataset, while a random subset of predictor variables is considered during node splitting. This dual randomization promotes diversity among the trees, thereby reducing model variance and minimizing the likelihood of overfitting. For regression applications, the final prediction is obtained by averaging the outputs of all constituent trees, resulting in a robust model with improved generalization performance and resistance to noise in the training data (27, 28).

Mathematically, the prediction of a Random Forest model for a regression problem can be expressed as (29):

$$\hat{y}=\frac{1}{N_{\mathrm{trees}}}\overset{N_{\mathrm{trees}}}{\underset{i=1}{\sum }}f_{i}(x)$$

In this context, the symbol $\hat{y}$ symbolizes the collective prediction produced by the ensemble of decision trees. $N_{\mathrm{trees}}$ corresponds to the total quantity of decision trees within the random forest, while $f_{i}(x)$ denotes the prediction generated by each individual decision tree, specifically the *i*-th tree.

Extremely Randomized Trees, often abbreviated as Extra Trees (ET), builds upon the concept of Random Forest by introducing even more randomness during tree construction. In Extra Trees, random thresholds for feature splits are selected, further reducing variance and enhancing generalization. The ensemble prediction is similar to Random Forest and involves averaging the predictions of individual trees (30).

Gradient Boosting is an ensemble method that constructs DTs sequentially. Each tree is designed to correct the errors made by the previous ones. It optimizes a loss function, often Mean Squared Error (MSE), through gradient descent (31, 32). The ensemble prediction in Gradient Boosting is a weighted sum of individual tree predictions (32, 33):

$$\hat{y}=\overset{N_{\mathrm{trees}}}{\underset{i=1}{\sum }}\alpha_{i}f_{i}(x)$$

Here, $\alpha_{i}$ represents the contribution of each tree, and $f_{i}(x)$ indicates the final prediction made by the *i*-th tree.

XGBoost is an efficient and regularized implementation of gradient-boosted decision trees. It constructs an ensemble sequentially, such that each newly added regression tree is trained to reduce the prediction errors of the current model. For an input vector $x_{i}$, the prediction generated by an ensemble containing *K* trees is expressed as (32, 34):

$${\hat{y}}_{i}=\overset{K}{\underset{k=1}{\sum }}f_{k}(x_{i}),\;f_{k}\in F,$$

where ${\hat{y}}_{i}$ is the predicted response for observation *i*, *K* is the total number of regression trees, $f_{k}$ denotes the function represented by the *k* th tree, and F is the space of possible regression-tree structures. At boosting iteration *t*, the prediction is updated as:

$${\hat{y}}_{i}^{\left(t\right)}={\hat{y}}_{i}^{\left(t-1\right)}+f_{t}(x_{i}).$$

The newly added tree is determined by minimizing a regularized objective function:

$$L^{\left(t\right)}=\overset{n}{\underset{i=1}{\sum }}l(y_{i},{\hat{y}}_{i}^{\left(t-1\right)}+f_{t}(x_{i}))+\Omega (\;f_{t}),$$

where *n* is the number of training observations, $y_{i}$ is the experimental response, l(⋅) is the training-loss function, and $\Omega (\;f_{t})$ penalizes the complexity of the newly added tree. The regularization term is defined as:

$$\Omega (\;f_{t})=\gamma T+\frac{\lambda }{2}\overset{T}{\underset{\;j=1}{\sum }}w_{j}^{2},$$

where *T* denotes the number of terminal leaves, $w_{j}$ is the prediction score assigned to leaf *j*, and γ and λ are regularization parameters controlling tree complexity and leaf-weight magnitude, respectively. By minimizing both the prediction loss and the complexity penalty, XGBoost improves the ensemble iteratively while limiting unnecessary model complexity and reducing the risk of overfitting (32, 35).

## 4 Results and discussion

The performance of four models—RF, ET, GB, and XGB—was assessed via a number of statistical metrics. For the prediction of Nystatin solubility, the models were assessed based on their R-squared (R^2^) scores, MAPE, and RMSE. Table 1 provides a summary of the numeric results for solubility estimation using these models to compare their fitting performance.

**Table 1:** Numeric results for solubility.

Model	R^2^ Score	MAPE	RMSE
RF	0.92894	0.2524	0.05622
ET	0.87827	0.2993	0.07754
GB	0.98142	0.0593	0.03415
XGB	0.96559	0.09698	0.04797

The risk of overfitting was carefully considered during model development for the solubility dataset. As shown in Table 1, the models achieved high (R^2^) values together with relatively low RMSE and MAPE values, with the GB model providing the best overall performance ((R^2^ = 0.98142), RMSE (=0.03415), and MAPE (=0.0593)). Nevertheless, these performance values alone cannot completely exclude overfitting. Therefore, the optimized ML models were evaluated using some unseen test data, and their hyperparameters were optimized using validation data to avoid risk of overfitting. The consistency of performance across the training, validation, and test datasets, particularly the absence of a substantial deterioration in test-set accuracy, indicates that the models maintained satisfactory generalization rather than merely fitting the training observations for solubility data. Accordingly, the reported results provide no clear evidence of substantial overfitting, although validation using additional independent solubility data at different *P* and T values would further strengthen this conclusion.

**Figure 2:**
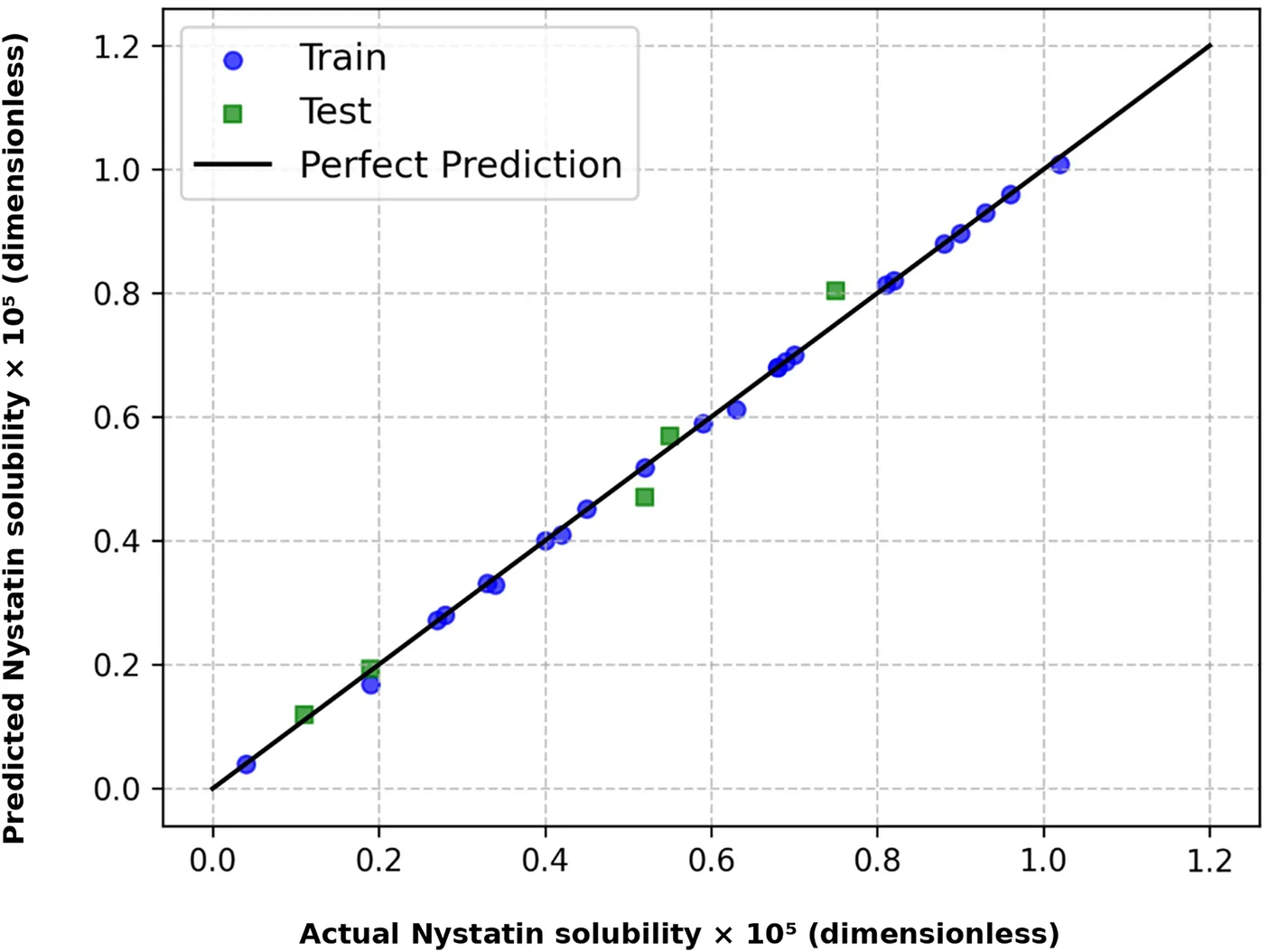
Predicted solubility values Vs. Observed solubility values.

Among these four models, Gradient Boosting (GB) stands out with an impressive score of 0.98142 in terms of R^2^, signifying a robust relationship between the input parameters and the prediction of Nystatin Solubility. Furthermore, GB showcases the lowest MAPE and RMSE values, underscoring its superior precision and accuracy in making predictions. Consequently, GB emerges as the greatest model for forecasting Nystatin solubility in the solvent. This model's performance in predicting solubility values is depicted in Figure 2 by comparing with the measured data. Meng and Liu (11) reported almost similar accuracy for their ML models in modeling Nystatin solubility.

For correlating the density data, the same evaluation metrics were used to assess the model performance. Table 2 shows the results for this output.

**Table 2:** Numeric results for density.

Model	R^2^ Score	MAPE	RMSE
RF	0.9375	0.0455	32.557
ET	0.95155	0.03398	30.722
GB	0.91339	0.066	52.904
XGB	0.90981	0.0593	49.413

**Figure 3:**
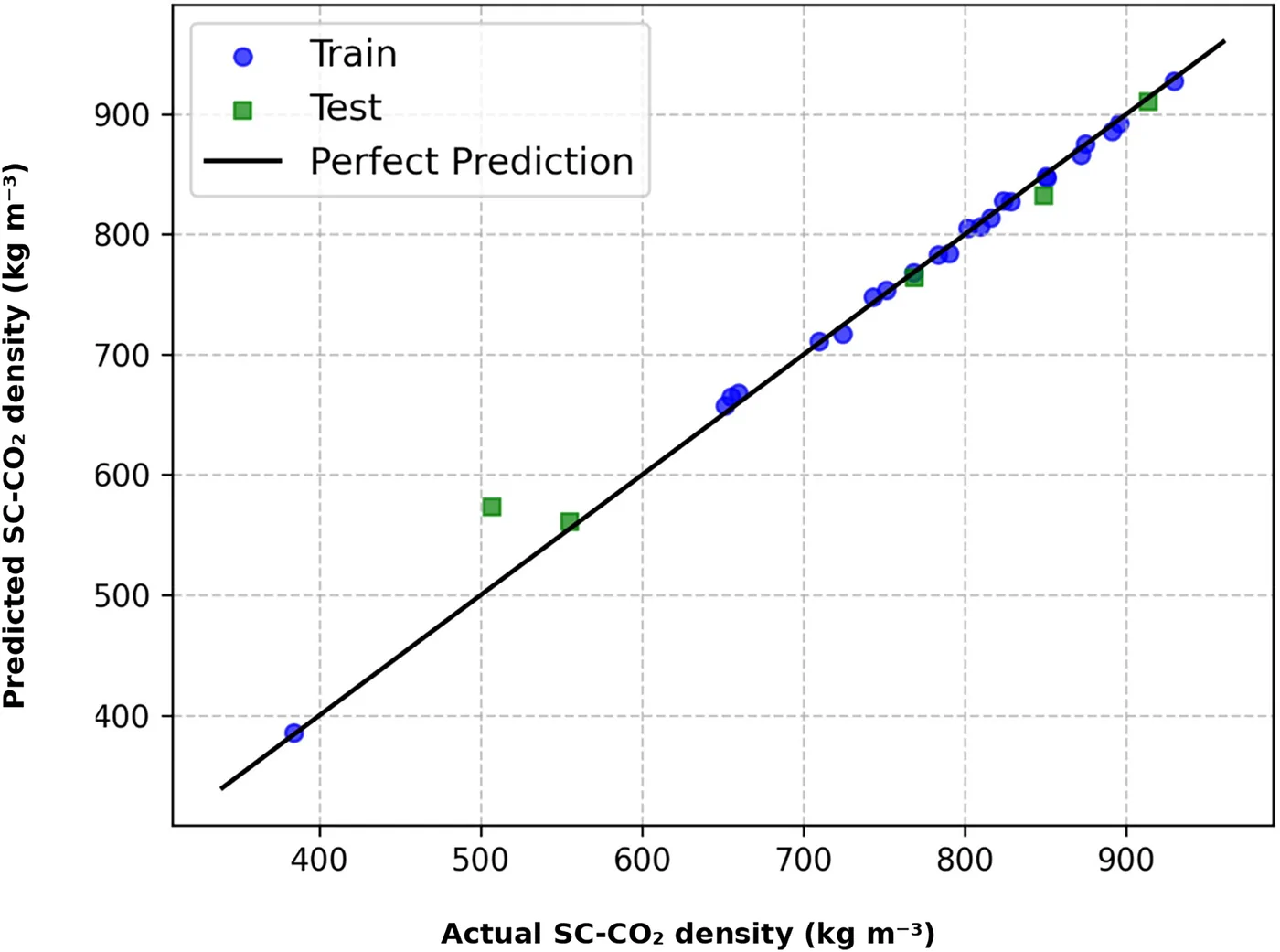
Predicted density values Vs. Observed density values.

**Figure 4:**
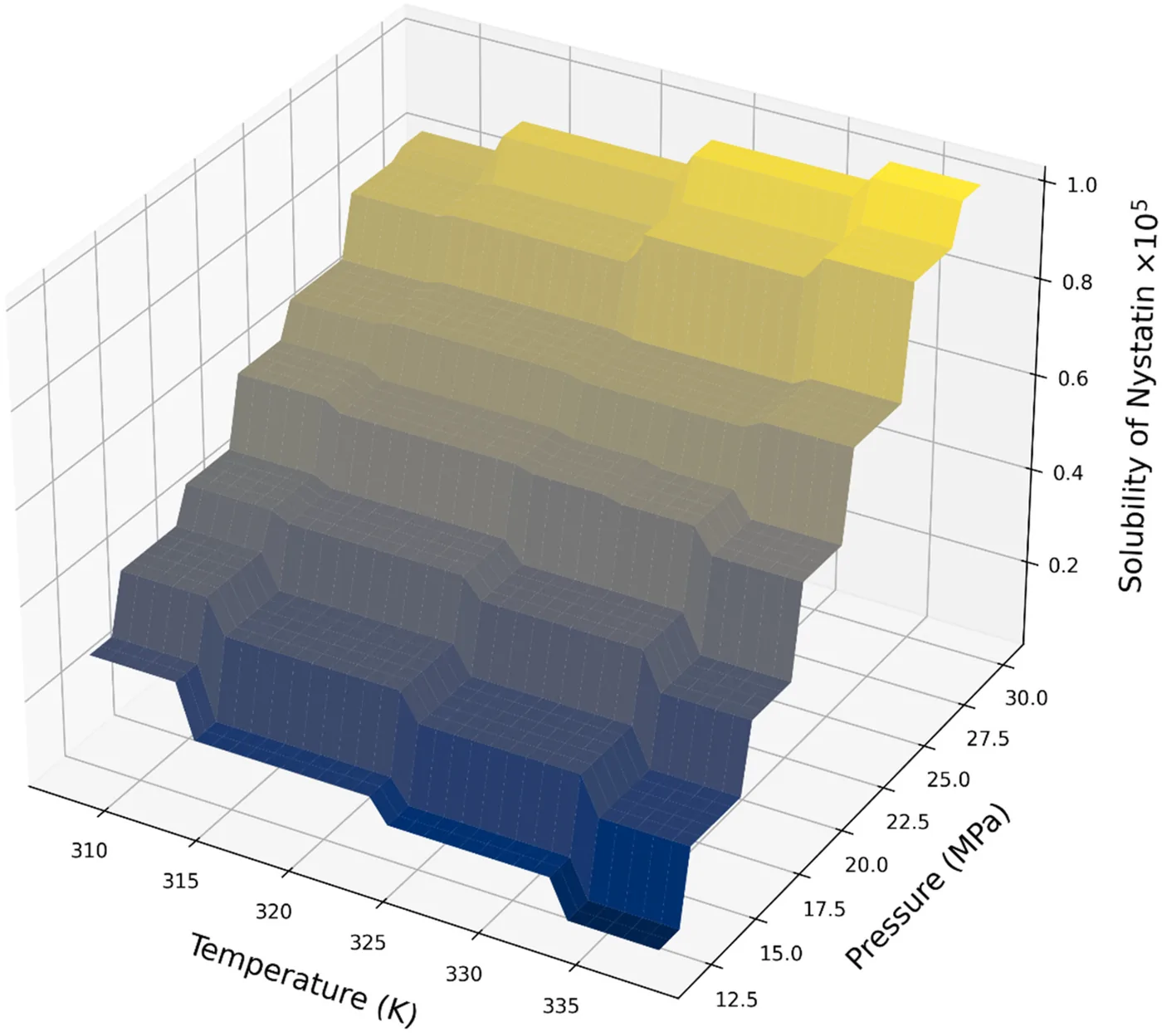
Predicted 3D surface of solubility.

**Figure 5:**
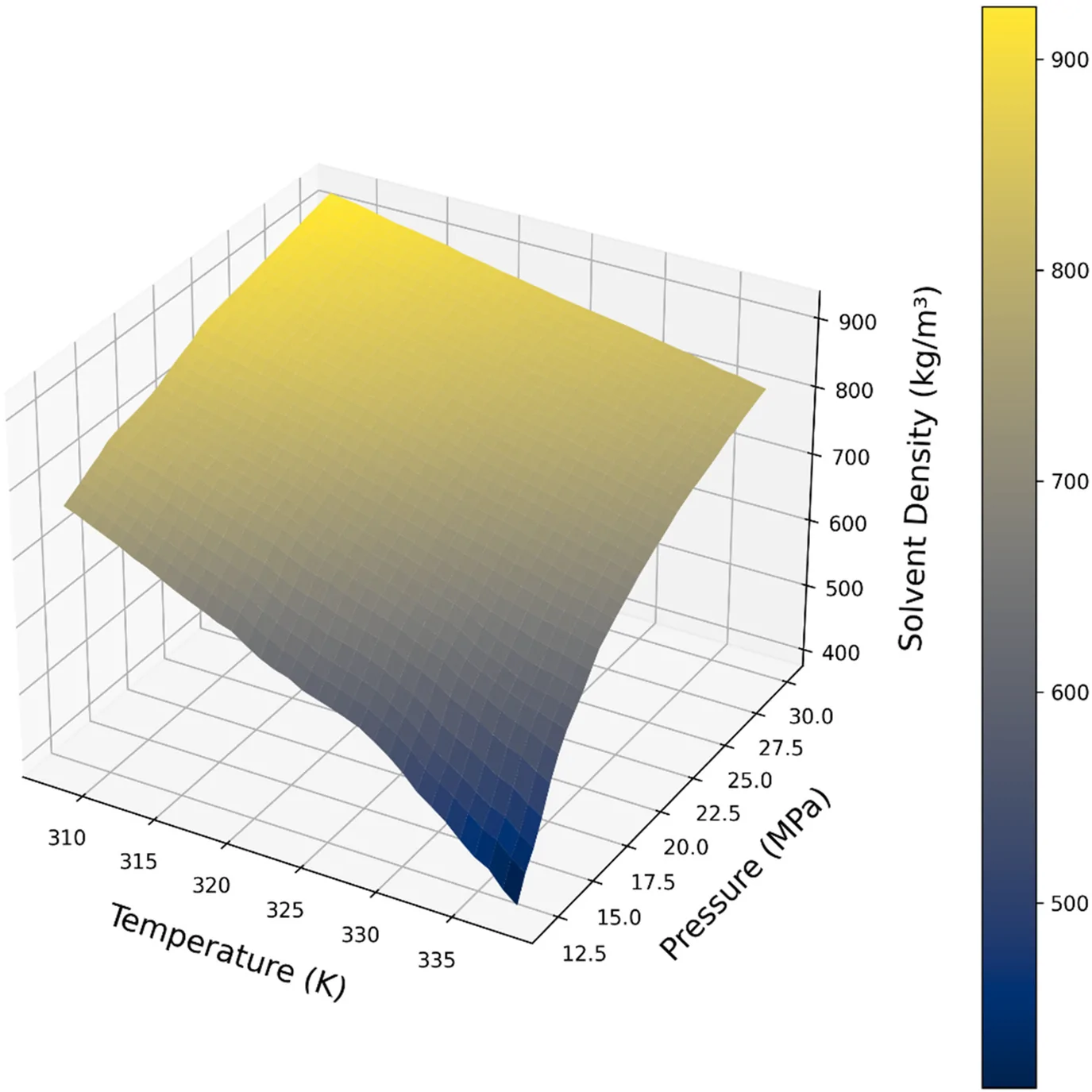
Predicted 3D surface of solvent density.

In the realm of predicting SC-CO_2_ density, ET model notably secured the highest R^2^ of 0.95155, revealing a strong and robust correlation between the input parameters and Density of solvent. Additionally, ET demonstrated the lowest MAPE and RMSE values, which attest to its remarkable precision and accuracy in making predictions. Hence, ET emerges as the most proficient model for forecasting SC-CO_2_ density. The visualization of the concordance between observed and predicted densities can be observed in Figure 3. Using GB for solubility of Nystatin and ET for density as the best-fit models one can visualize both outputs as the functions of input features which are shown as 3D plots in Figures 4, 5. The results can be compared with those reported by Meng and Liu (11) and Zhang (16), specifically for density which shows similar 3D surface trend.

**Figure 6:**
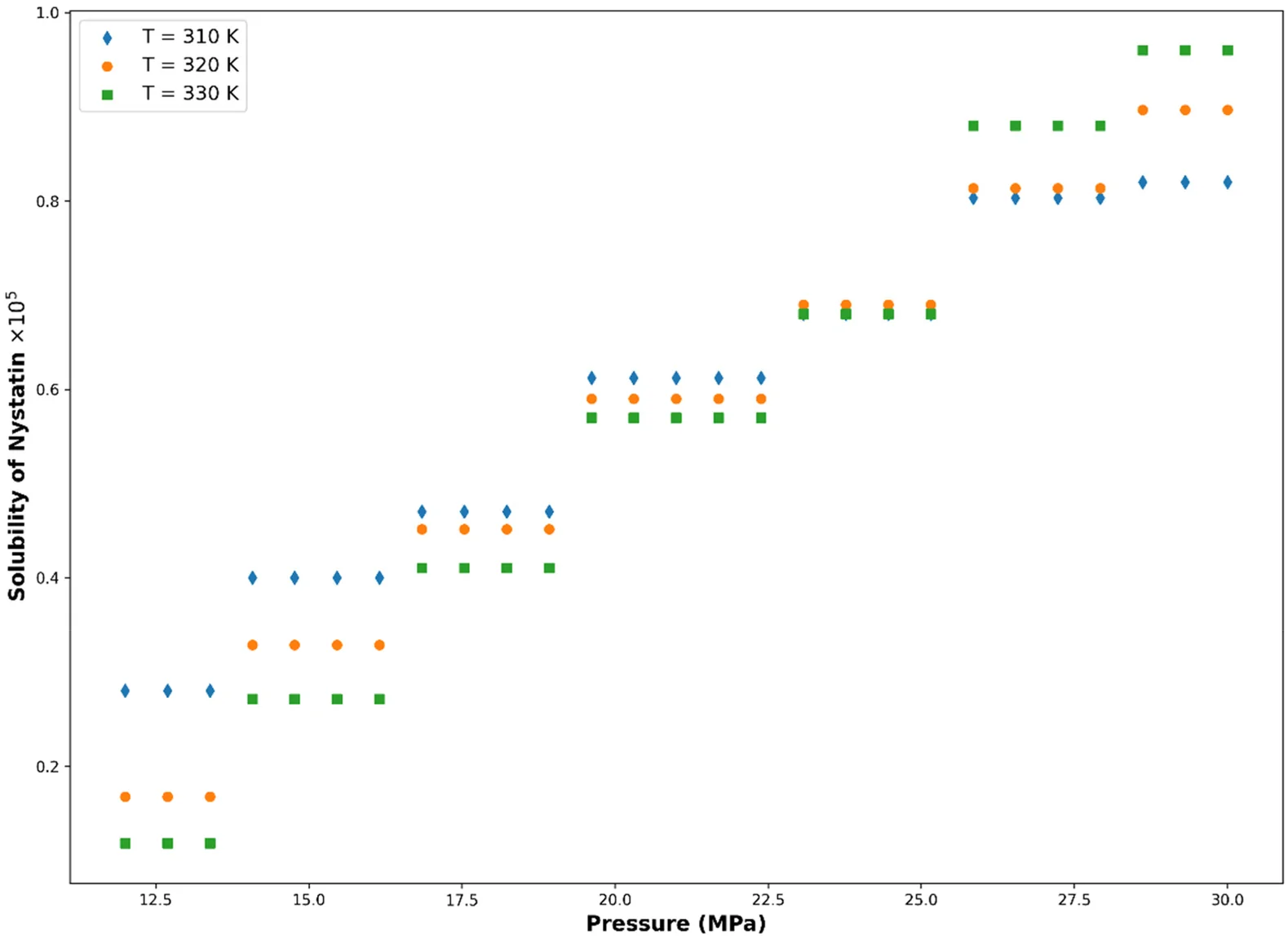
Pressure effect on solubility, captured using the model.

**Figure 7:**
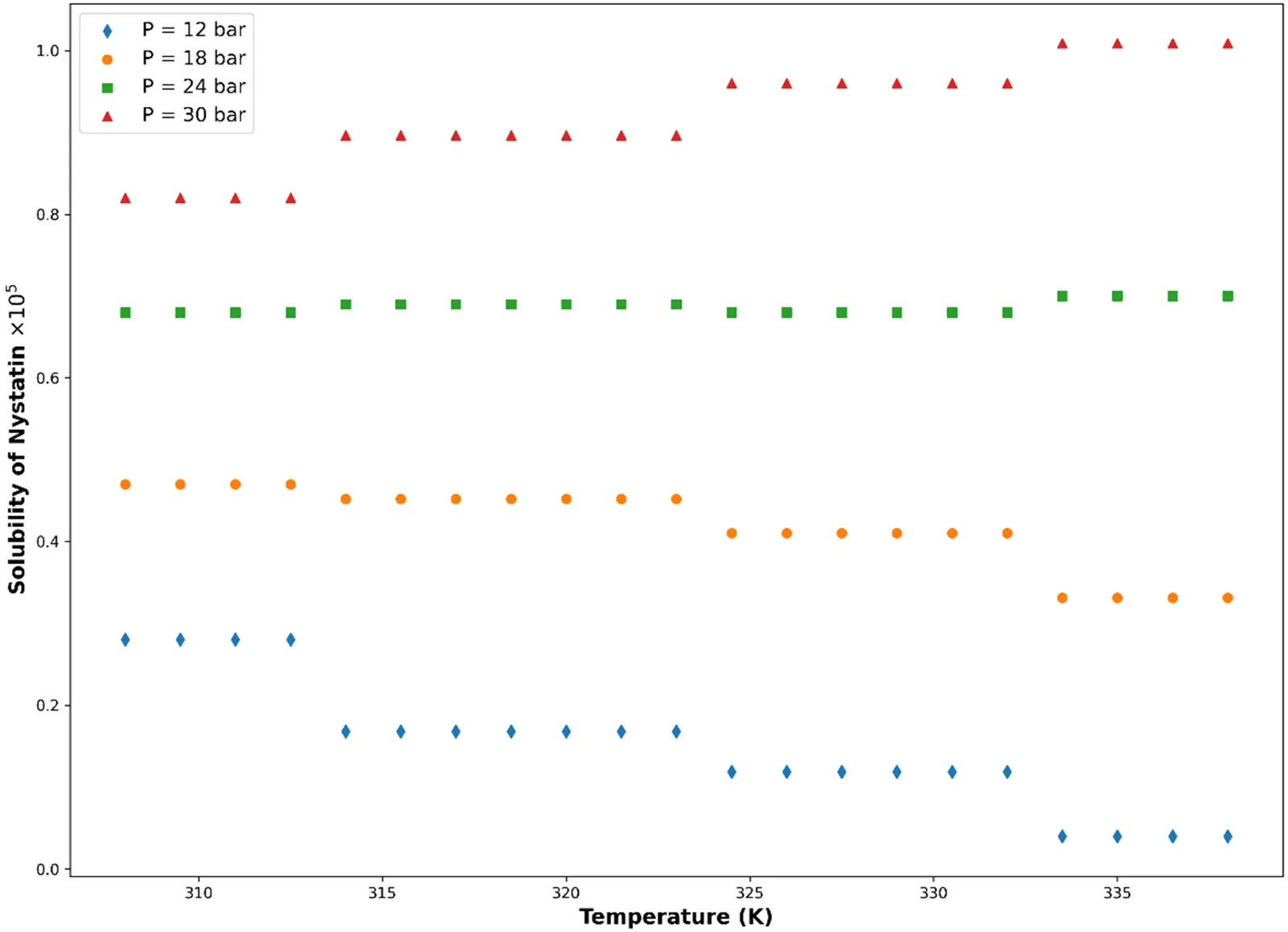
Temperature effect on solubility, captured using the model.

**Figure 8:**
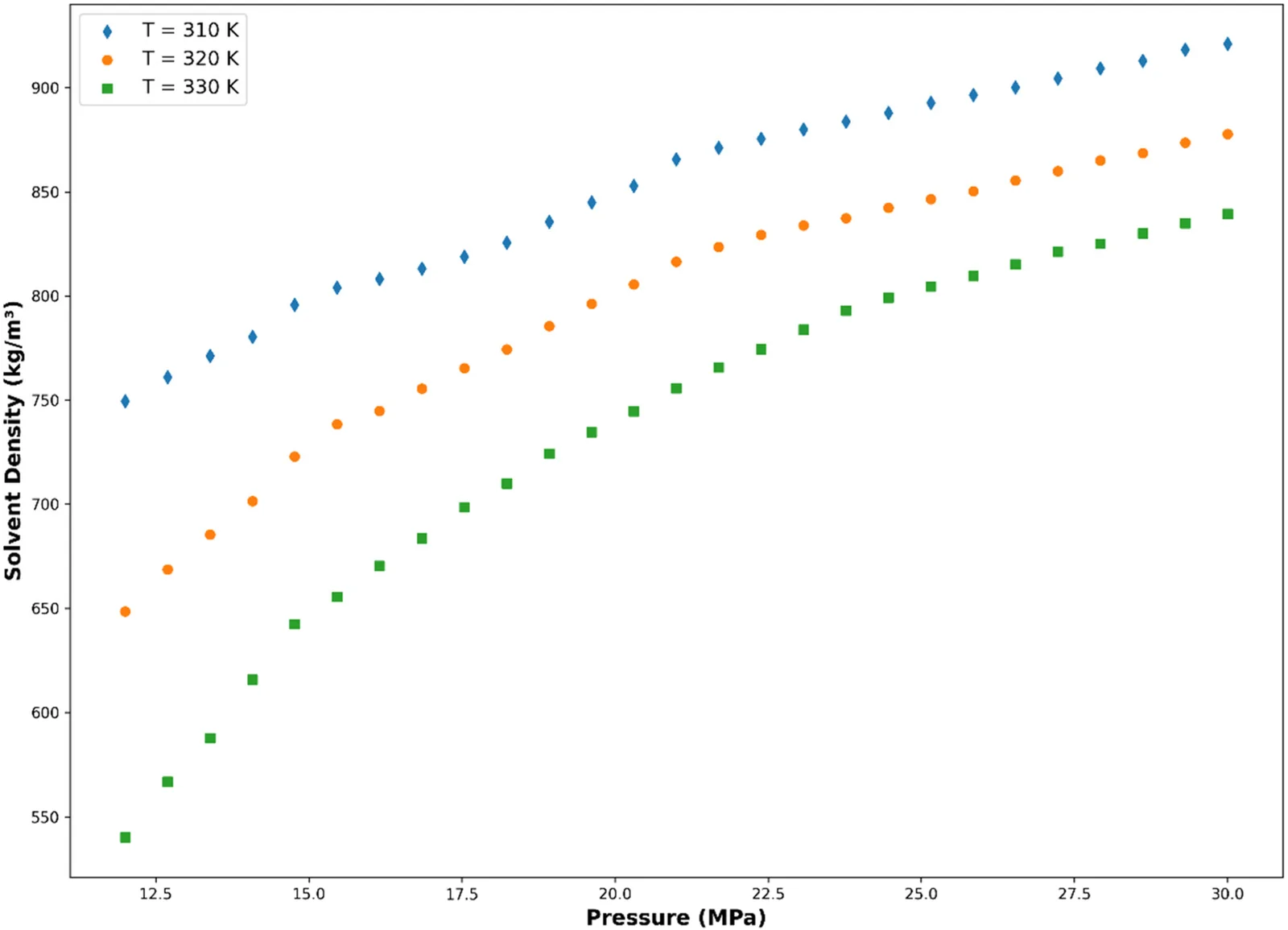
Pressure effect on density, captured using the model.

Finally, partial dependence of inputs on target outputs are shown in Figures 6–8. It is notable that the pressure would directly increase the drug solubility, while the temperature would do the same at higher pressure values. At relatively low pressures, an increase in temperature leads to a reduction in drug solubility, which can primarily be attributed to the characteristic crossover-pressure phenomenon observed in supercritical fluid systems. The results of ML modeling reported by Zhang (16) for Nystatin indicated the same behavior for this drug at various *P* & T values.

Figure 6 illustrates the combined effects of pressure and temperature on the solubility of Nystatin. At all investigated temperatures, solubility generally increases with increasing pressure, demonstrating that pressure is the dominant factor governing the solubility of drug in the supercritical solvent. This behavior can be attributed to the increase in fluid density and solvent power at elevated pressures, which enhances solute–solvent interactions (11). At approximately 23–25 MPa, the solubility values at the three temperatures become comparable, indicating a crossover region. Beyond this region, particularly above approximately 26 MPa, increasing temperature enhances solubility, with the highest value observed at 330 K and 28–30 MPa.

As indicated in Figure 8, increasing pressure significantly enhances SC-CO₂ density across the entire temperature range. At 12 bar, the solvent density decreases considerably from approximately 770 to 385 kg/m^3^ as temperature increases, whereas substantially higher densities are maintained at elevated pressures (16). For example, at 30 bar, the density remains above 800 kg/m^3^ throughout the investigated temperature range, demonstrating the dominant role of pressure in compressing the supercritical fluid and increasing molecular packing (11, 16). This observation has been also reported and confirmed by Zhang (16) for Nystatin.

These results further justify the incorporation of temperature and pressure as key input variables in machine learning models developed for predicting drug solubility in SC-CO_2_ systems, as variations in these parameters directly modify the solvent environment and consequently influence drug dissolution behavior.

Furthermore, the developed machine learning models can be employed as predictive tools for optimizing the solubility process by identifying operating conditions that maximize solubility, particularly with respect to pressure and temperature as the main input features. Although increasing pressure and temperature may enhance solubility of Nystatin within the investigated operating range (T and P), such improvements should be evaluated alongside their economic implications in terms of drug production costs. Higher operating pressures and temperatures generally require greater energy consumption and may consequently increase processing and equipment costs. Therefore, a comprehensive optimization framework should incorporate both solubility performance and techno-economic considerations to determine operating conditions that provide an appropriate balance between enhanced solubility and process efficiency.

## 5 Conclusion

The solubility of Nystatin and the density of SC-CO_2_ in relation to changes in temperature and pressure was investigated by computational techniques. Leveraging machine learning models, including RF, ET, GB, and XGB, and employing the Tabu Search (TS) algorithm for hyper-parameter tuning, valuable insights were gained into the predictive capabilities of these models.

For Nystatin solubility prediction, the GB model emerged as the most accurate, achieving an impressive R^2^ of 0.98142, as well as the lowest MAPE and RMSE. This suggests that GB is well-suited for accurate predictions of Nystatin solubility.

Regarding SC-CO_2_ density prediction, Random Forest (RF) and Extremely Randomized Trees (ET) demonstrated robust performance, with R^2^ value of 0.9375 and 0.95155, respectively. Both models exhibited low MAPE and RMSE values, signifying their ability to accurately forecast SC-CO_2_ density across different temperature and pressure settings.

In conclusion, this research has provided valuable tools and insights for predicting essential properties of SC-CO_2_, which has wide-ranging applications in industries such as pharmaceuticals and materials science. The accurate prediction of Nystatin solubility and SC-CO_2_ density under varying conditions is crucial for optimizing processes and product development. The machine learning models utilized in this study offer practical solutions for these predictions, contributing to more efficient and precise industrial processes. The major limitation for building data-driven models is the small dataset for solubility in supercritical CO_2_ which cannot make a robust model. Future work may involve further refinement of these models and their application in real-world industrial settings to enhance process design and optimization.

## Data Availability

The original contributions presented in the study are included in the article/Supplementary Material, further inquiries can be directed to the corresponding author/s.
